# A Multichannel Solid-State Potentiometric Sensor Array for Heavy Metal Ions

**DOI:** 10.3390/s26103003

**Published:** 2026-05-10

**Authors:** Zongfeng Wei, Guanliang Li, Zhuqing Wang, Shicai Xu, Enguang Lv, Weiwei Yue

**Affiliations:** 1 School of Communication and Electronic Engineering, Shandong Normal University, Jinan 250014, China; zfwei2026@163.com; 2 Institute of Biophysics, Dezhou University, Dezhou 253023, China; dzulgl@163.com (G.L.); wangzq128@163.com (Z.W.); shicaixu@dzu.edu.cn (S.X.)

**Keywords:** multichannel potentiometric array, peptide recognition, simultaneous detection, heavy metal ions

## Abstract

Heavy metal ions are common environmental contaminants that threaten aquatic ecosystems and human health. However, conventional analytical techniques often require expensive instrumentation and complex sample pretreatment. This work presents a peptide-based multichannel solid-state potentiometric microelectrode array for simultaneous detection of Cu^2+^, Cd^2+^, and Pb^2+^. The array consists of one shared Ag/AgCl reference electrode and three groups of gold indicator microelectrodes (10 μm in diameter), with each group containing three parallel electrodes for replicate measurements. Each group is functionalized with a distinct peptide receptor specific to one target metal ion. The proposed array exhibits near-Nernstian responses and good selectivity against common interfering ions. Practical applicability is demonstrated by analyzing spiked lake water samples under a fixed chloride condition, with recoveries ranging from 94% to 105%. This work provides a miniaturized, reproducible, and versatile platform for simultaneous potentiometric detection of multiple heavy metal ions, and the peptide-based recognition strategy can be extended to other targets by simply replacing the peptide sequences.

## 1. Introduction

Heavy metal ions are persistent environmental pollutants mainly released from industrial effluents, mining activities, agricultural runoff, and domestic wastewater [1,2]. Unlike organic pollutants, these metal ions cannot be degraded and tend to accumulate in sediments and living organisms [3]. For example, chronic exposure to Cu^2+^ can cause liver and kidney damage [4], while Pb^2+^ and Cd^2+^ are known neurotoxins and carcinogens, respectively [5,6]. Therefore, rapid, sensitive, and simultaneous monitoring of these metal ions in natural water bodies is of critical importance for environmental protection and public health. Traditional analytical techniques such as atomic absorption spectroscopy (AAS) and inductively coupled plasma mass spectrometry (ICP-MS) could realize the simultaneous determination of multi metal ions, and offer excellent sensitivity and accuracy [7,8]. However, they generally require expensive instrumentation, trained personnel, complex sample pretreatment, and laboratory settings [9,10].

Potentiometric analysis, as one important branch of electrochemical analysis, shows the advantages of low cost, ease of minimization, and rapid response. It has been widely used in environmental monitoring, medical diagnosis, food analysis and other fields [11]. However, conventional potentiometric analysis is typically restricted to single-analyte detection, which greatly limits its practical applicability. To overcome this limitation and achieve simultaneous detection of multiple targets, considerable efforts have been made. A series of polymeric membrane-based potentiometric sensors were developed for the simultaneous detection of several species [12]. Mou et al. reported on a magneto-controlled potentiometric method. By combining with deep learning algorithms, the technique could be used for the classifying and quantifying diverse small molecules [13]. However, their method was demonstrated on small-molecule analytes rather than ionic species. Jasiński et al. [14] and Niemiec et al. [15,16] developed potentiometric multi-sensor systems for the simultaneous detection of common ions (e.g., Na^+^, K^+^, Ca^2+^, Cl^−^) rather than heavy metal ions. Researchers also have explored other signal readout modes based on the potential change to realize the simultaneous detection of multi targets. Ding et al. developed a simple, versatile, and sensitive platform for simultaneous chronopotentiometric detection of two molecules with a single polymeric membrane ion-selective electrode. Under a series of periodic galvanostatic polarization, the electrode was used for the detection of two opposite charged ions [17]. Fan et al. designed a peptide-based electrical array sensor employing the current density ratios as the readout for discriminating multiple heavy metal ions (Cu^2+^, Zn^2+^, Cd^2+^, Mn^2+^, and Co^2+^) [18]. Bakker’s group reported an optical sensing approach based on a potentiometric sensing array, allowing simultaneous detection of multiple ions [19,20]. However, these methods suffer from limitations such as complex polarization steps [17], reliance on current-density ratios rather than direct potential readout [18], or the need for external optical components [19,20], which compromise the inherent simplicity of potentiometric sensing. Meanwhile, these approaches for simultaneous detection of multiple targets are mainly based on polymeric liquid membrane electrodes. Although these methods exhibit good sensitivity, selectivity, and practical applicability, they still suffer from common limitations of polymeric liquid membrane electrodes, including instability caused by leakage of internal components from the polymeric membrane and a complex preparation process [21].

Solid-state potentiometric sensors with the advantages of good stability and ease of minimization and preparation were also developed for the simultaneous determination of multi-targets. Gismera et al. reported a solid-state potentiometric sensor array for simultaneous quantification of heavy metals (Cu^2+^, Pb^2+^, Cd^2+^, and Zn^2+^) [22]. However, the use of non-specific electrodes results in poor selectivity, which limits practical applications in complex environmental samples. Xu et al. constructed a self-calibrated potentiometric sensor array based on peptide recognition for the simultaneous detection of two foodborne pathogens [23]. However, the approach simply combined two independent electrodes rather than forming an integrated multichannel array. To the best of our knowledge, no previous study has reported a solid-state potentiometric sensor array for simultaneous detection of heavy metal ions. A thorough literature search revealed that this area remains largely unexplored. This is mainly because most reported solid-state electrodes are of conventional size with relatively high detection limits. In addition, the lack of highly selective recognition elements limits their development.

In this paper, a multichannel solid-state potentiometric sensor array for heavy metal ions is proposed. The array is fabricated on a silicon wafer using standard photolithography, metal deposition, and lift-off processes, yielding one shared Ag/AgCl reference electrode and three groups of gold indicator microelectrodes (each group containing three parallel 10 μm diameter electrodes). The microelectrode geometry enhances mass transport and minimizes background current, thereby achieving a significantly lower detection limit. This design enables replicate measurements and simultaneous potentiometric readout for three target ions. To achieve selective recognition, different peptides are immobilized onto each group of indicator electrodes via gold-thiol self-assembly. Peptides offer high specificity toward metal ions, as well as chemical stability, facile synthesis, and easy surface modification [24]. Consequently, the proposed peptide-functionalized microelectrode array allows simultaneous, sensitive, and selective detection of Cu^2+^, Pb^2+^, and Cd^2+^, and its practical applicability is successfully validated in lake water samples.

## 2. Materials and Methods

### 2.1. Chemicals and Materials

Silver (99.99%) and gold (99.99%) evaporation pellets, AZ5214 positive photoresist, AZ400K developer, silicon wafer, and the designed photomasks in this study were purchased from Suzhou Research Materials Microtech Co., Ltd. (Suzhou, China). All peptide sequences (Cu^2+^-selective peptide [25]: MPA-Gly-Gly-His; Cd^2+^-selective peptide [26]: MPA-His-Ser-Gln-Val-Lys-Phe; Pb^2+^-selective peptide [27]: Cys-Asp-Arg-Val-Tyr-Ile-His-Pro-Phe-His-Leu) were derived from the literature and synthesized by GL Biochem (Shanghai, China) with purity > 95%. Tris(2-carboxyethyl)phosphine hydrochloride (TCEP), Trizma base, Cu(NO_3_)_2_, Cd(NO_3_)_2_, Pb(NO_3_)_2_, and other interfering ions were obtained from Sigma-Aldrich (St. Louis, MO, USA). Ultrapure water (18.2 MΩ·cm) was used throughout. Lake water samples were collected from Zhixin Lake in Dezhou University (Dezhou, China) and stored at 4 °C before analysis.

### 2.2. Design of Photomasks

**Figure 1:**
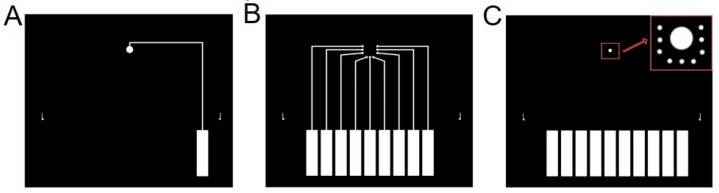
Photomasks used for (**A**) reference electrode patterning, (**B**) indicator electrodes patterning, and (**C**) encapsulation.

Three photomasks were used for the fabrication of microelectrode array. Figure 1A shows the pattern for reference electrode (Ag/AgCl), in which the disk electrode has a diameter of 1200 μm. Figure 1B shows the pattern for the indicator electrodes (Au), with each disk electrode having a diameter of 400 μm. Figure 1C shows the final encapsulation pattern. After encapsulation, the exposed reference electrode diameter is 600 μm, and the exposed indicator electrode diameter is 10 μm. Narrow connecting lines were prone to breakage during the metal lift-off step, leading to a significant decrease in fabrication yield. Therefore, the width of both the gold and silver connecting lines was set to 200 μm. One alignment mark is placed on each side of the mask to enable precise alignment during lithography. Ten rectangular contact regions (2 mm × 8 mm) were designed for wire connection during potentiometric measurement.

### 2.3. Fabrication of the Microelectrode Array

**Figure 2:**
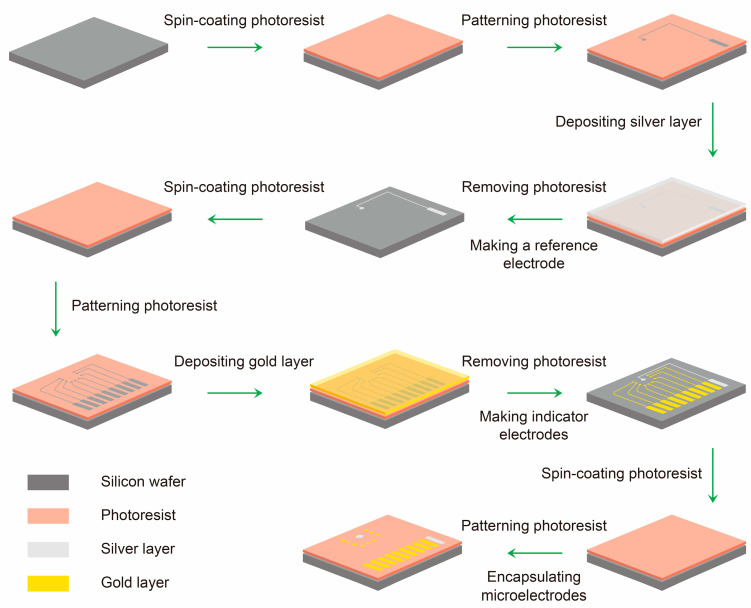
Schematic illustration of the fabrication process of the multichannel solid-state potentiometric microelectrode array.

The microelectrode array was fabricated on a 2-inch single-side polished silicon wafer (500 μm thickness). The wafer was cleaned sequentially with acetone, ethanol, and ultrapure water in an ultrasonic bath (60 kHz, 10 min each) and dried in a vacuum oven at 60 °C for 2 h. All photolithography steps were performed using a URE-2000/35 deep ultraviolet lithography system (Chinese Academy of Sciences, Beijing, China). The detailed fabrication process of the sensor array is shown in Figure 2.

#### 2.3.1. Reference Electrode Patterning

AZ5214 positive photoresist was spin-coated onto the wafer at 2000 rpm for 5 s followed by 4000 rpm for 20 s (spin coater: WH-SC-01, Suzhou, China). The thickness of the photoresist layer was measured to be 1.47 μm (Figure S1) using a Surfcorder ET 150 (Kosaka Laboratory Ltd., Tokyo, Japan). The wafer was soft-baked at 95 °C for 4 min, then exposed through a photomask containing the reference electrode pattern (Figure 1A) for 10 s. After exposure, the wafer was developed in AZ400K developer (1:4 *v*/*v* in water) for 30 s. Then, a silver layer (40 nm thick) was deposited by the high-vacuum resistive evaporation system (0.1 Å/s, VZZ-300, VNANO Vacuum Technology Co., Ltd., Beijing, China), followed by a 2 h vacuum standing period. Lift-off was carried out in acetone (immersing for 5 min) to remove the remaining photoresist and the unpatterned silver, leaving the patterned silver layer.

#### 2.3.2. Indicator Electrode Patterning

A second photoresist layer was spin-coated and patterned using a photomask with the indicator electrode pattern (Figure 1B) under identical lithography conditions. After development, a gold layer (40 nm thick) was deposited by resistive evaporation at 0.1 Å/s, followed by 2 h vacuum standing. Lift-off in acetone yielded three groups of gold indicator microelectrodes (each group containing three parallel 10-μm-diameter electrodes).

#### 2.3.3. Encapsulation

**Figure 3:**
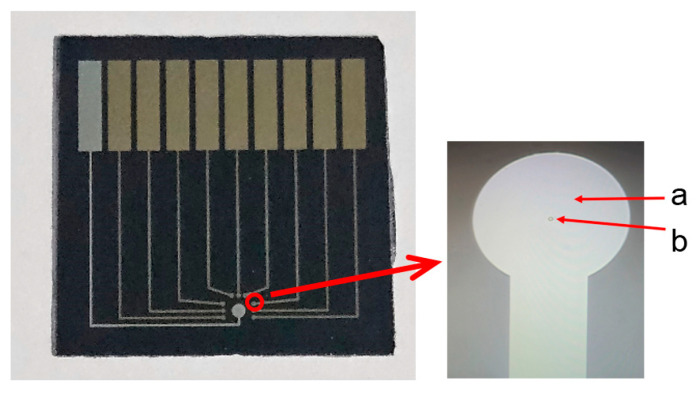
Optical images of the multichannel solid-state potentiometric sensor array. (a) Areas covered by photoresist, which serve as the passivation layer; (b) Magnified view of the gold microelectrodes (10 μm disk diameter). The array consists of one shared Ag/AgCl reference electrode (center) and three groups of gold indicator microelectrodes (each group contains three parallel 10-μm-diameter disks). The rectangular contact regions are coated with conductive silver epoxy for electrical connection.

A third photoresist layer was spin-coated and patterned using an encapsulation mask (Figure 1C). After development, only gold electrodes (10 μm diameter), silver electrodes (600 μm diameter) and the rectangular contact regions (2 mm × 8 mm) were exposed, while other region including the connecting lines was covered by the photoresist. As shown in Figure 3, the metal disk diameter is significantly larger than the final gold indicator electrode region encapsulated with photoresist. This design not only enables precise control of the electrode dimensions but also minimizes alignment errors. The final microelectrode array consisted of one Ag reference electrode (later chlorinated to Ag/AgCl) and three groups of Au indicator electrodes (nine Au disks in total). Thus, the multichannel electrode array was successfully fabricated (Figure 3). Prior to use, conductive silver epoxy was applied to the rectangular contact regions of the array to enable further experiments.

### 2.4. Surface Modification of Indicator Electrodes with Peptides

Three different peptide sequences (each dissolved in 10 mM Tris-HCl buffer, pH 7.0, containing 1 mM TCEP to reduce disulfide bonds) were prepared at a concentration of 10 μM. For each group of indicator electrodes, 5 μL of the corresponding peptide solution was dropped onto the electrode surface and incubated for 10 min. This deposition–incubation cycle was repeated three times to complete the modification. The Cu^2+^-selective peptide was immobilized on group I, the Cd^2+^-selective peptide on group II, and the Pb^2+^-selective peptide on group III. The peptide could be adsorbed on the surface of the electrode via Au-S bond. After incubation, the modified electrodes were thoroughly rinsed with water and ready to use. For comparison with conventional-sized electrodes (3 mm diameter), we measured the potentiometric responses of conventional electrodes modified with the same peptides to different metal ions.

### 2.5. Potentiometric Measurements

All potentiometric measurements were performed at room temperature using a 16-channel potentiometric readout system (EMF 16 interface, Lawson Labs Inc., Malvern, PA, USA). The shared Ag reference electrode was chlorinated by applying 5 μL of 1 mM FeCl_3_ in 0.1 M KCl, incubating for 10 min, and repeating three times, yielding an Ag/AgCl reference electrode. For each measurement, 20 μL of standard solution or sample was dropped onto the sensor array. Potentials were recorded simultaneously from all nine indicator electrodes (three groups × three parallel electrodes) versus the Ag/AgCl reference electrode. After each concentration change, the potential was monitored until stabilization (about 100 s), and the steady-state potential was recorded for subsequent analysis.

Calibration curves were obtained by adding aliquots of standard metal ion solutions (Cu^2+^, Pb^2+^, or Cd^2+^) into a background electrolyte solution (10 mM Tris-HCl buffer, pH 7.0). The constant chloride ion concentration in Tris-HCl buffer ensures the stability of the Ag/AgCl reference electrode. Four concentration points ranging from 1.0 × 10^−7^ M to 1.0 × 10^−4^ M (1.0 × 10^−7^, 1.0 × 10^−6^, 1.0 × 10^−5^, and 1.0 × 10^−4^ M) were measured in logarithmic increments. For each concentration, three replicate measurements (using the three parallel electrodes within each group) were performed, and the average potential ± standard deviation (SD) was reported. The limit of detection (LOD) was calculated as three times the standard deviation of the blank divided by the slope of the calibration curve (3σ).

Selectivity coefficients (log Kᵢ,ⱼ) were evaluated using the separate solution method. Potentials of each peptide-modified electrode were measured separately in solutions containing 1.0 × 10^−5^ M of the primary ion (i) and in solutions containing 1.0 × 10^−5^ M of each interfering ion (j). The coefficients were calculated using the Nikolskii–Eisenman equation.

### 2.6. Lake Water Sample Analysis

Prior to measurement, NaCl was added to each sample to achieve a final Cl^−^ concentration of 10 mM in order to stabilize the potential of the Ag/AgCl reference electrode. The samples were then spiked with standard solutions of Cu^2+^, Cd^2+^, and Pb^2+^ at two concentration levels: 1.0 × 10^−7^ M and 1.0 × 10^−6^ M. The spiked samples were analyzed using the peptide-based sensor array following the same potentiometric procedure described in Section 2.5. The concentration of each metal ion was determined using calibration curves constructed from measurements of Cu^2+^, Pb^2+^, and Cd^2+^ standards in 10 mM NaCl. Recovery (%) was calculated as (measured concentration/spiked concentration) × 100. Each spiked sample was measured in triplicate using the three parallel electrodes within each group.

## 3. Results

### 3.1. Optimization of the Thickness of Au Layer

Gold is a noble metal, and a uniform thin film can typically be formed when the thickness reaches approximately 110 Å. Therefore, the initial gold layer thickness was set to 110 Å. However, field emission scanning electron microscopy (FE-SEM) images (Figure S2) revealed numerous cracks on the gold film surface at this thickness. When the thickness was increased to 220 Å, the cracks were significantly reduced. Further increasing the thickness to 400 Å resulted in a completely crack-free, uniform, and dense gold film morphology. Therefore, a gold film thickness of 400 Å was selected for the fabrication of the microelectrode array.

### 3.2. Characterization of Ag, Au Layer

To analyze the surface morphology and thickness characteristics of the silver and gold layers, atomic force microscopy (AFM, Bruker Multimode 8) was employed, and the results are shown in Figure S3. The Ra value represents the surface roughness of the metal layer. The average surface roughness measured at three different positions on the silver layer and the gold layer was 1.07 ± 0.05 nm and 0.94 ± 0.07 nm, respectively. This indicates that uniform, dense, and tightly packed Ag and Au layers were obtained. Meanwhile, the thickness of the Ag and Au layers measured by AFM was approximately 400 Å (Figure S4), which is consistent with the deposition settings of the vacuum evaporation system.

### 3.3. Characterization of the Silver Microelectrode

The surface morphology and elemental distribution of the Ag/AgCl microelectrode were characterized using FE-SEM, and the results are shown in Figure S5A. The microelectrode surface was covered with square-shaped particles, which were identified as redundant AgCl nanoparticles. Energy dispersive spectrometer (EDS) analysis (Figure S5B) revealed a small amount of iron on the Ag/AgCl microelectrode surface, which is likely attributable to residual FeCl_3_ solution from the chlorination step. Apart from this trace residue, no other impurities were detected, confirming the successful fabrication of the Ag/AgCl reference electrode.

### 3.4. Characterization of the Gold Indicator Microelectrode

**Figure 4:**
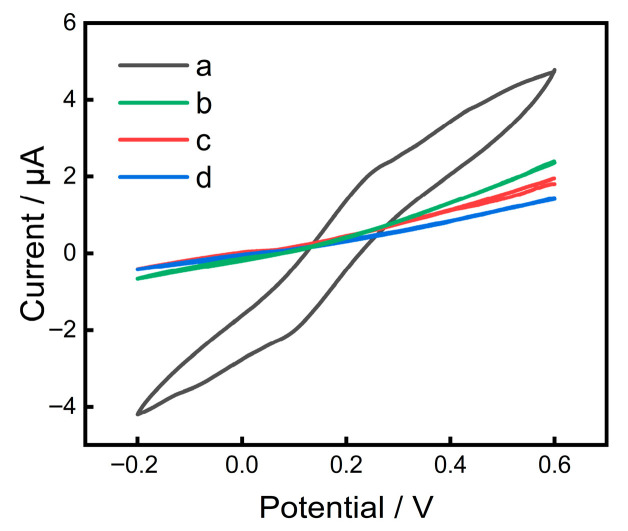
Cyclic voltammograms of different peptide-modified electrodes recorded at 50 mV/s in 0.1 M KCl containing 5 mM Fe(CN)_6_^4−/3−^: (a) bare gold microelectrode, and gold microelectrode modified with (b) Cu^2+^-selective peptide, (c) Cd^2+^-selective peptide, (d) Pb^2+^-selective peptide.

To confirm successful peptide immobilization on the electrode surface, cyclic voltammetry (CV), EDS analysis, and contact angle test were performed. As shown in Figure 4, the bare gold microelectrode exhibits the highest peak current. After modification with peptides, the peak current decreases significantly, confirming successful peptide immobilization. Owing to the extremely small surface area, the working currents of the microelectrode are typically in the nA-μA range. Meanwhile, EDS analysis (Figure S6) showed that the bare gold electrode contained only Au, whereas the peptide-modified electrodes exhibited additional S, N, O, and P signals, further verifying the successful immobilization of the peptide layers. Water contact angle measurements were performed to verify the successful immobilization of peptides onto the gold electrode surfaces. As shown in Figure S7, the bare gold surface exhibited a contact angle of 89.28°, indicating its hydrophobic nature. After modification with the Cu^2+^-selective peptide, the contact angle dramatically decreased to 28.79°, confirming the attachment of a highly hydrophilic peptide layer. The Cd^2+^-selective and Pb^2+^-selective peptides gave contact angles of 54.53° and 66.68°, respectively. The variation among the three peptides is attributed to differences in their amino acid compositions and hydropathies. These results provide direct evidence that the peptides are successfully immobilized on the gold surfaces via self-assembly.

### 3.5. Detection of Heavy Metal Ions

**Figure 5:**
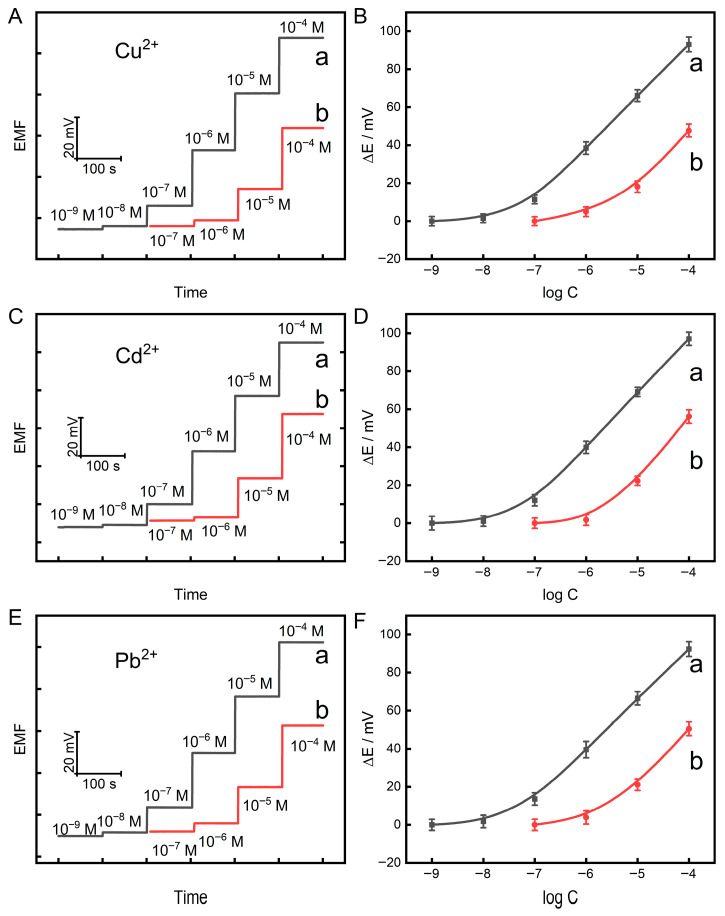
Potential responses to heavy metal ions (**A**,**C**,**E**) at different concentrations with the developed sensor array (a) and the peptide-modified gold electrodes with a diameter of 3 mm (b). Calibration curves (**B**,**D**,**F**) for heavy metal ions with the developed sensor array (a) and the peptide-modified gold electrodes with a diameter of 3 mm (b). The real-time potentiometric response curves were normalized for comparative analysis. Error bars represent standard deviations from three parallel electrodes.

As can be seen from Figure 5 (a), the potential gradually increases with increasing heavy metal ion concentration. The relationship between the steady-state potential and the logarithm of the ion concentration was fitted by linear regression, yielding slopes of 27.22 mV/decade for Cu^2+^, 26.31 mV/decade for Cd^2+^, and 28.45 mV/decade for Pb^2+^. The corresponding regression equations are: ΔE (mV) = 27.22 log[Cu^2+^] + 168.5, ΔE (mV) = 26.31 log[Cd^2+^] + 202.0, ΔE (mV) = 28.45 log[Pb^2+^] + 197.7. The limits of detection (LOD) are 2.9 × 10^−8^ M for Cu^2+^, 3.2 × 10^−8^ M for Cd^2+^, and 3.0 × 10^−8^ M for Pb^2+^, (3σ). The linear range spans from 1.0 × 10^−7^ M to 1.0 × 10^−4^ M. Conventional-sized electrodes (3 mm diameter) modified with the same peptides can also be used for the detection of heavy metal ions (Figure 5 (b)). However, the developed solid-state potentiometric microelectrode array shows better sensitivity. This improvement is primarily attributed to two factors. First, the small diameter (10 μm) of the microelectrodes enables radial diffusion, which enhances mass transport efficiency. Second, the reduced double-layer capacitance and background current lead to a higher signal-to-noise ratio.

### 3.6. Stability of the Developed Array

**Figure 6:**
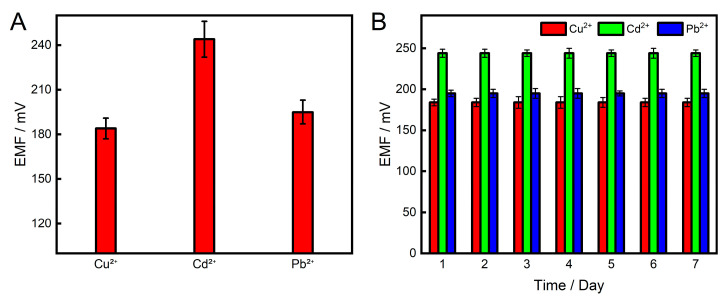
(**A**) Potentiometric responses of three independently fabricated sensor chips to 10 μM Cu^2+^, Cd^2+^, and Pb^2+^. Error bars represent the standard deviation of three parallel measurements (*n* = 3). (**B**) Stability of the peptide-modified sensor array to 10 µM of Cu^2+^, Cd^2+^, and Pb^2+^ within a 7-day period.

To evaluate the reproducibility between different sensor chips, three independently fabricated microelectrode arrays were tested for their potentiometric response to 10 μM of Cu^2+^, Cd^2+^, and Pb^2+^. Figure 6A shows a bar chart of the average potentials ± SD for each metal ion across the three chips. The relative standard deviations (RSDs) of the measured potentials were all below 5% (*n* = 3 for each metal ion), demonstrating good device-to-device reproducibility, which is attributed to the consistent photolithographic microfabrication process.

The long-term stability of the peptide-modified sensor array was evaluated by storing a fabricated chip at 4 °C in 10 mM Tris-HCl buffer (pH 7.0) for 7 days. Daily measurements of the potential responses to 10 μM Cu^2+^, Cd^2+^, and Pb^2+^ were taken. As shown in Figure 6B, the potential values for each metal ion remained relatively stable throughout the 7-day period, with no obvious loss of response.

### 3.7. Selectivity of the Array

Selectivity is a critical parameter for potentiometric sensors, especially when analyzing environmental samples containing multiple interfering ions. The potentiometric responses to different interfering ions were measured (Figure S8), and the selectivity coefficients (log Kᵢ_,_ⱼ) for each peptide-modified electrode were evaluated using the separate solution method. As listed in Table 1, all three peptide-functionalized electrodes exhibited excellent selectivity for their respective target ions over the tested interferents.

### 3.8. Sample Detection

The practical applicability of the sensor array was evaluated by analyzing spiked lake water samples after adding NaCl to a fixed Cl^−^ concentration of 10 mM (to ensure a stable reference electrode potential). As shown in Table 2, the recoveries for Cu^2+^, Pb^2+^, and Cd^2+^ at spiked concentrations of 1.0 × 10^−7^ M and 1.0 × 10^−6^ M ranged from 94% to 105%, with relative standard deviations (RSDs) below 7% for triplicate measurements (three parallel electrodes per group). These results demonstrate that the sensor array can reliably detect heavy metal ions in real lake water matrices under the fixed-chloride condition. The satisfactory recoveries and good reproducibility confirm the accuracy and robustness of the proposed method.

## 4. Discussion

In this work, we developed a peptide-based multichannel solid-state potentiometric microelectrode array for simultaneous detection of Cu^2+^, Cd^2+^, and Pb^2+^. The sensor array exhibits near-Nernstian responses, detection limits of about 3.0 × 10^−8^ M for each ion, excellent selectivity, and good recoveries (94–105%) in spiked lake water samples.

A key contribution of this work is the successful miniaturization of the sensor array using standard photolithography and physical vapor deposition techniques. These methods allow precise control over the size and shape of the microelectrodes. By replacing manual assembly with photolithographic patterning, we significantly reduced manual fabrication errors and improved device-to-device reproducibility. The three-parallel-electrode design per target ion further enhances measurement reliability by providing built-in replicates on a single chip.

This work demonstrates that peptide-functionalized solid-state potentiometric microelectrode arrays can serve as a versatile platform for simultaneous multi-ion detection. The design principles could be extended to other analytes by simply changing the recognition peptides. Indeed, numerous peptide sequences have been reported for clinical diagnostics, food safety, and environmental monitoring [28,29]. Therefore, through straightforward substitution of the peptide probes, the present platform holds promise for future extension to these fields.

## Figures and Tables

**Table 1:** Logarithmic selectivity coefficients of peptide-modified array.

Interfering Ion (j)	log K_Cu,j_ (Cu^2+^-Selective)	log K_Pb,j_ (Pb^2+^-Selective)	log K_Cd,j_ (Cd^2+^-Selective)
Cu^2+^	-	−2.5 ± 0.1	−3.3 ± 0.1
Pb^2+^	−3.1 ± 0.1	-	−3.2 ± 0.1
Cd^2+^	−3.2 ± 0.2	−2.2 ± 0.2	-
Mg^2+^	−3.5 ± 0.1	−3.1 ± 0.1	−4.0 ± 0.1
Zn^2+^	−3.8 ± 0.2	−3.8 ± 0.2	−3.6 ± 0.2
Ca^2+^	−3.7 ± 0.3	−3.6 ± 0.3	−3.5 ± 0.3

**Table 2:** Standard addition recoveries of Cu^2+^, Cd^2+^, and Pb^2+^ in spiked lake water samples (*n* = 3).

Metal Ion	Spiked (mol/L)	Measured (mol/L)	Recovery (%)
Cu^2+^	1.0 × 10^−7^	0.96 × 10^−7^	96
	1.0 × 10^−6^	1.03 × 10^−6^	103
Cd^2+^	1.0 × 10^−7^	1.05 × 10^−7^	105
	1.0 × 10^−6^	0.98 × 10^−6^	98
Pb^2+^	1.0 × 10^−7^	0.94 × 10^−7^	94
	1.0 × 10^−6^	1.01 × 10^−6^	101

## Data Availability

The original contributions presented in this study are included in the article. Further inquiries can be directed to the corresponding authors.

## Supplementary Materials

The following supporting information can be downloaded at: https://www.mdpi.com/article/10.3390/s26103003/s1, Figure S1: The thickness of the photoresist layer measured by Surfcorder ET 150; Figure S2: Field emission scanning electron microscopy images of Au layer with different thicknesses; Figure S3: Atomic force microscopy images and corresponding height profiles of the metal layers; Figure S4: Atomic force microscopy cross-sectional images and corresponding height profiles of the metal layers; Figure S5: Scanning electron microscopy images and element analysis of the Ag/AgCl electrode; Figure S6: EDS analysis of bare gold microelectrode and peptide-modified gold electrodes; Figure S7. Contact angle measurements of bare gold layer, and gold layer modified with different peptides. Figure S8: Potentiometric responses of the peptide-modified electrodes to target ions and interfering ions.

## Abbreviations

The following abbreviations are used in this manuscript:

AAS	Atomic absorption spectroscopy
AFM	Atomic force microscopy
EDS	Energy dispersive spectrometer
FE-SEM	Field emission scanning electron microscopy
ICP-MS	Inductively coupled plasma mass spectrometry
MPA	3-Mercaptopropionic acid
SD	Standard deviation
TCEP	Tris(2-carboxyethyl)phosphine hydrochloride
