# Supplementary material for: A Multichannel Solid-State Potentiometric Sensor Array for Heavy Metal Ions
Source: Sensors (Basel). 2026 May 10;26(10):3003. doi: 10.3390/s26103003 (PMC13210780; doi:10.3390/s26103003)
Supplement: Supplementary file 1 [file sensors-26-03003-s001.zip › sensors-4290276-supplementary.pdf]

Supplementary Materials for:

A Multichannel Solid-state Potentiometric Sensor Array for Heavy Metal Ions

Zongfeng Wei [1], Guanliang Li [2], Zhuqing Wang [2], Shicai Xu [2], Enguang Lv [2,*] and Weiwei Yue [1,*]

[1] School of Communication and Electronic Engineering, Shandong Normal University, Jinan 250014, China; zfwei2026@163.com (Z.W.)

[2] Institute of Biophysics, Dezhou University, Dezhou 253023, China; dzulgl@163.com (G.L.); wangzq128@163.com (Z.W.); shicaixu@dzu.edu.cn (S.X.)

* Correspondence: eglv@dzu.edu.cn (E.L.); yuewei@sdnu.edu.cn (W.Y.)

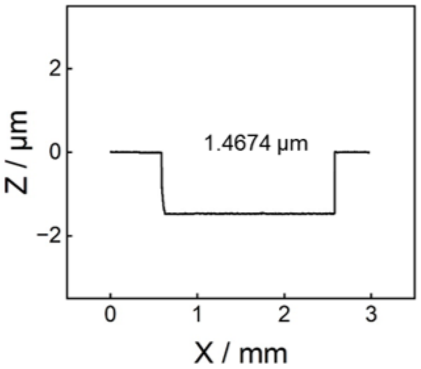

Figure S1. The thickness of the photoresist layer measured by Surfcorder ET 150.

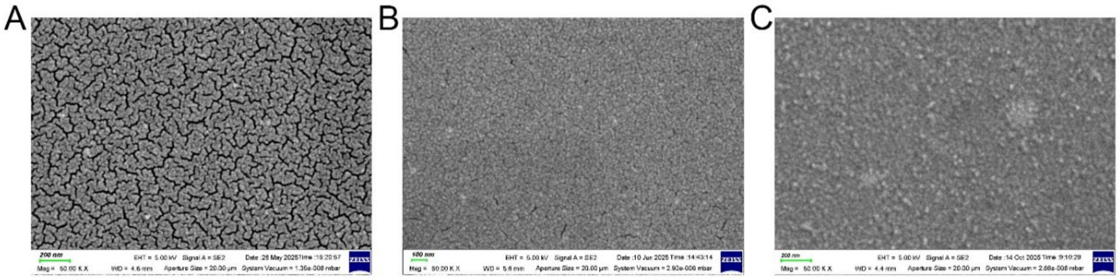

Figure S2. Field emission scanning electron microscopy images of Au layer with different thicknesses: (A) 110 Å, (B) 220 Å, and (C) 400 Å.

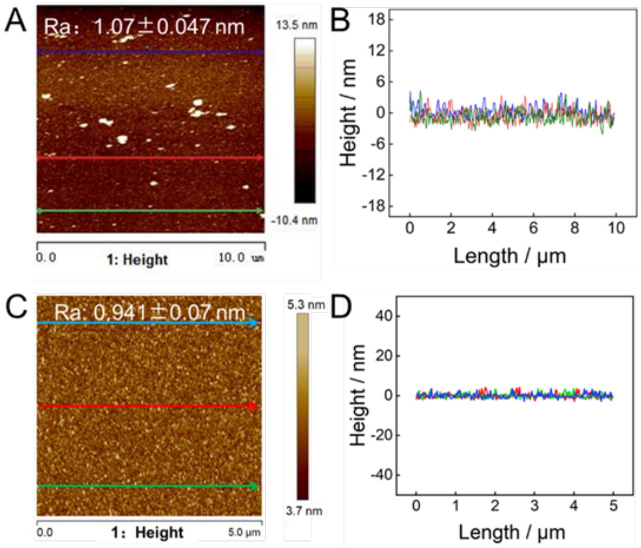

Figure S3. Atomic force microscopy images and corresponding height profiles of the metal layers. Surface morphologies of (A) silver and (C) gold films, respectively. Height fluctuations of (B) silver and (D) gold films along the blue, red, and green lines indicated in (A) and (C), respectively.

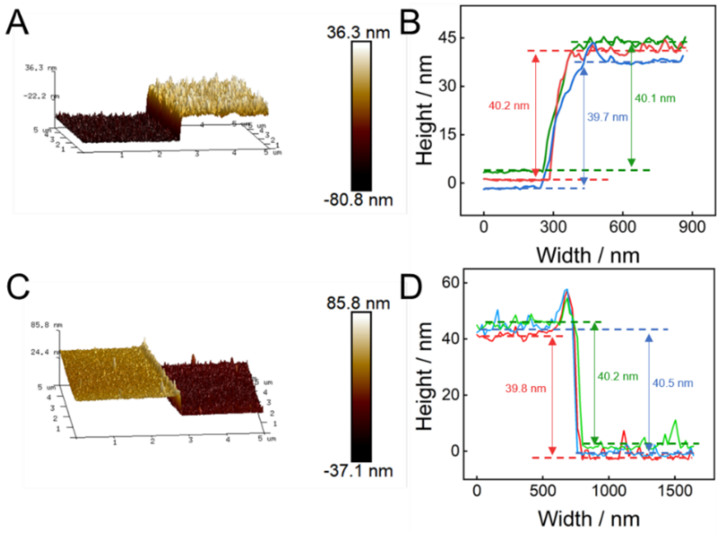

Figure S4. Atomic force microscopy cross-sectional images of (A) silver layer and (C) gold layer and corresponding height profiles of (B) silver layer and (D) gold layer.

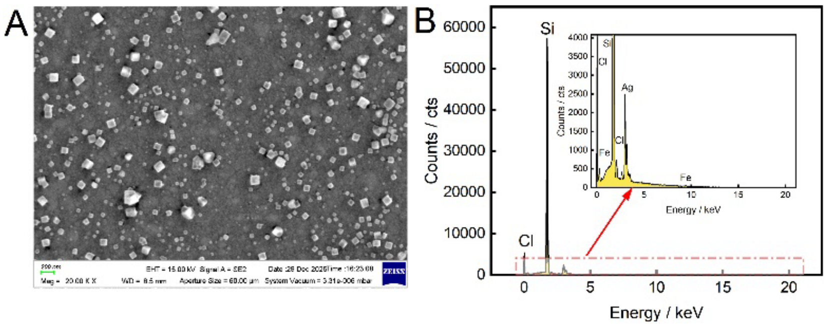

Figure S5. (A) Scanning electron microscopy images and (B) element analysis of the Ag/AgCl electrode surface.

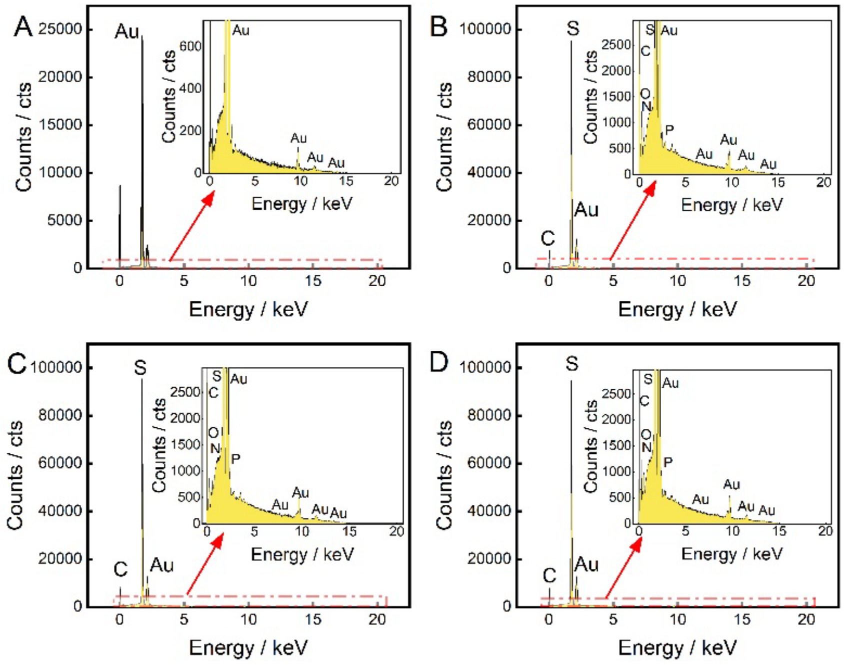

Figure S6. Energy dispersive spectrometer analysis of (A) bare gold microelectrode and peptide-modified gold electrodes: (B) Cu^{2+}-selective peptide, (C) Cd^{2+} selective peptide, and (D) Pb^{2+} selective peptide.

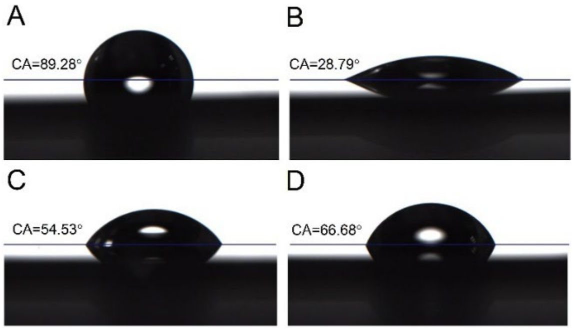

Figure S7. Contact angle measurements of (A) bare gold layer, and gold layer modified with (B) Cu^{2+}-selective peptide, (C) Cd^{2+}-selective peptide, and (D) Pb^{2+}-selective peptide.

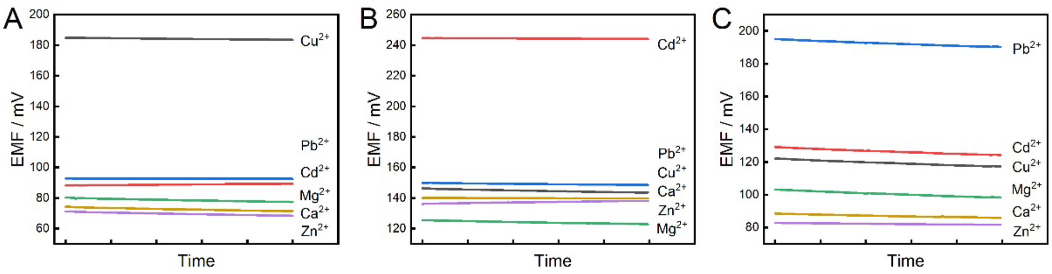

Figure S8. Potentiometric responses of different peptide-modified electrodes to target ions (Cu^{2+}, Cd^{2+}, Pb^{2+}) and interfering ions (Mg^{2+}, Zn^{2+}, Ca^{2+}): (A) Cu^{2+}-selective peptide-modified electrodes, (B) Cd^{2+}-selective peptide-modified electrodes, and (C) Pb^{2+}-selective peptide-modified electrodes.
